# Towards the Synthesis of Inosine Building Blocks for the Preparation of Oligonucleotides with Hydrophobic Alkyl Chains Between the Nucleotide Units †

**DOI:** 10.3390/molecules14114326

**Published:** 2009-10-26

**Authors:** Karl Köstler, Helmut Rosemeyer

**Affiliations:** Organische Materialchemie und Bioorganische Chemie, Institut für Chemie, Fachbereich Biologie/Chemie, Universität Osnabrück, Barbarastr. 7, D-49069 Osnabrück, Germany

**Keywords:** lipophilic oligonucleotides, phosphoramidites, nucleoside ketals

## Abstract

The scientific objective of the research reported in this manuscript was the synthesis of novel phosphoramidite building blocks for the preparation of lipophilic oligonucleotides. Reaction of inosine (**4**) with 4-oxopentyl-4-methylbenzoate (**2c**) in the presence of triethyl orthoformate and 4M HCl in 1,4-dioxane gave a diastereoisomeric mixture of the ketals **5**. Subsequent 4,4’-dimethoxytritylation at the 5’-hydroxyl afforded (R)-**6** + (S)-**6 **which could be separated chromatographically. Detoluoylation gave compounds (R)-**7 **and (S)-**7**. Phosphitylation of a diastereoisomeric mixture of **7** led to a mixture of four diastereoisomers of the corresponding 2-cyanoethylphosphoramidites **8**.

## Introduction

**Figure 1:**
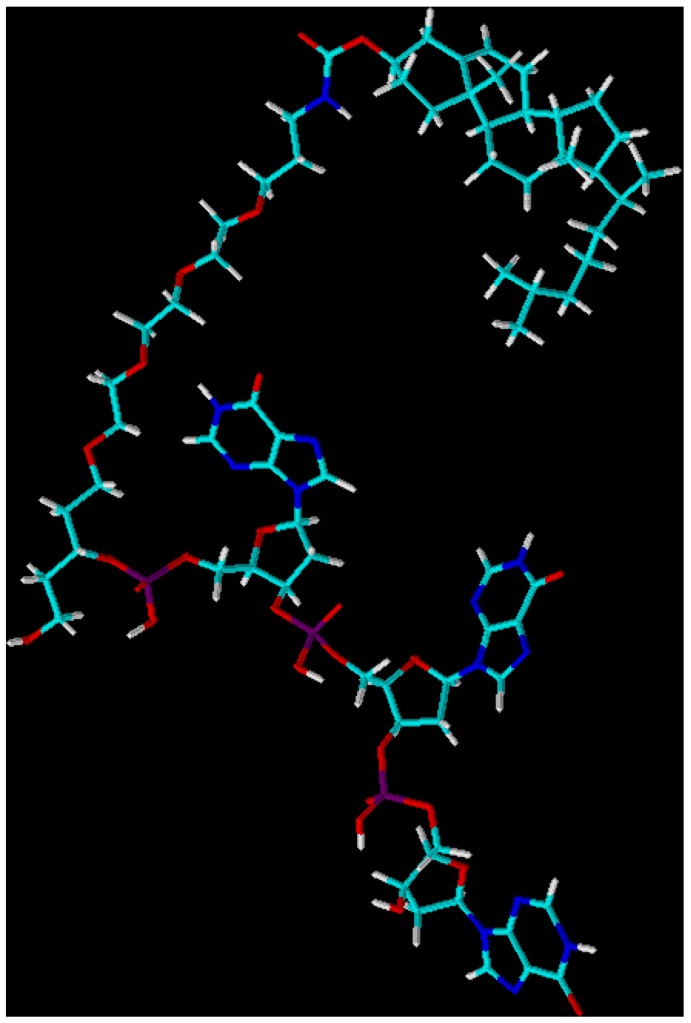
3D-Optimized structure of a 5’-cholesterol labelled trimer [5’-Chol-5’d(IpIpI)] using *ChemSketch*, 3D viewer, version 12.0 (Advanced Chemistry Developments, Inc. Toronto, Canada, http://www.acdlabs.com).

The low cell membrane permeability of oligonucleotides is one major drawback of antisense gene therapy. Therefore, as early as the eighties antisense and antigene oligomers were prepared which carried lipophilic groups, mostly at the termini. Other lipophilic modifications comprise derivatization of: (i) the nucleobases, (ii) of the glyconic residues or (iii) of the phosphodiester moiety of the oligonucleotides with hydrophobic side groups [1]. Of particular importance for the stability and activity of antisense oligonucleotides proved to be their modification with terminal cholesterol residues [2] which culminated in the development of the so-called “antagomirs” which are constructed from oligo(2’-OMe-ribonucleotides) carrying several phosphorothioate linkages at both termini and one cholesterol residue at the 5’-end. Such therapeutics have been successfully used for the *in-vivo* silencing of microRNAs [3]. The various applications of “nucleolipids” (nucleoside, nucleotide and oligonucleotide-based amphiphiles) were reviewed recently [4]. Appending a cholesterol moiety to an oligonucleotide, however, renders it so hydrophobic that its solubility in aqueous solvents is significantly reduced and its handling becomes problematic [5]. For example, a trimer consisting of three wobble nucleotides 2’-deoxyinosine [5’-d(IpIpI)] exhibits a calculated log*P* value of -6.67 ± 1.06 while its 5’-cholesterol derivative shown in Figure 1 exhibits a log*P* of +1.79 ± 1.27. 

**Figure 2:**
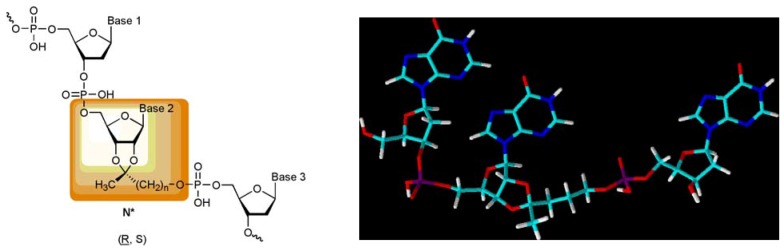
*Left*: General structure of an oligonucleotide with a lipophilic internucleotide linkage. *Right*: 3D-Optimized structure of a trimer [5’-d(IpI*pI)] with an inosine O-2’,3’-ketal with three methylene groups in the central position of the oligomer.

We, therefore, designed an oligonucleotide building block on the basis of an O-2’,3’-cyclic ketal – first with hypoxanthine as wobble base – which allows a stepwise hydrophobization of an oligonucleotide and which can be incorporated into all positions of a growing nucleic acid chain by conventional solid-phase synthesis yielding a general structure shown in Figure 2. In this manuscript we report first steps toward the preparation of this new kind of oligonucleotide building block.

## Results and Discussion

**Figure 3:**
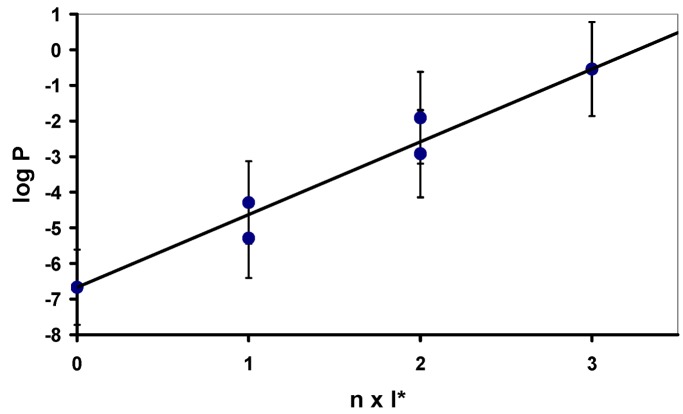
Calculatedlog *P* values of inosine oligonucleotide trimers as a function of the number of inosine O-2’,3’-cyclic ketal derivatives (see Figure 2).

In order to get an impression about the hydrophobization effect of the incorporation of one or more O-2’,3’-cyclic ketal derivative of inosine , I*, (with three CH_2_ groups in the ketal side chain) into an oligonucleotide, we first calculated the log*P* values [6] of a series of inosine trimers with varying substitutions of inosine by inosine ketals (I*) with a chain length of three methylene groups. Figure 3 and Table 1 display the increase of lipophilicity with the increasing number of modifications as well as the influence of the position of incorporation. 

**Figure 4:**
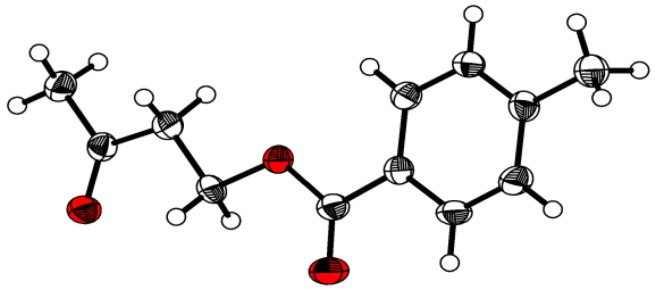
Ball and stick model of compound **2b** (with the exception of the hydrogen atoms, which are represented by use of spheres with a common isotropic radius, all other atoms are represented as thermal displacement ellipsoids showing 50% of the probability of the corresponding atom.

**Scheme 1:**
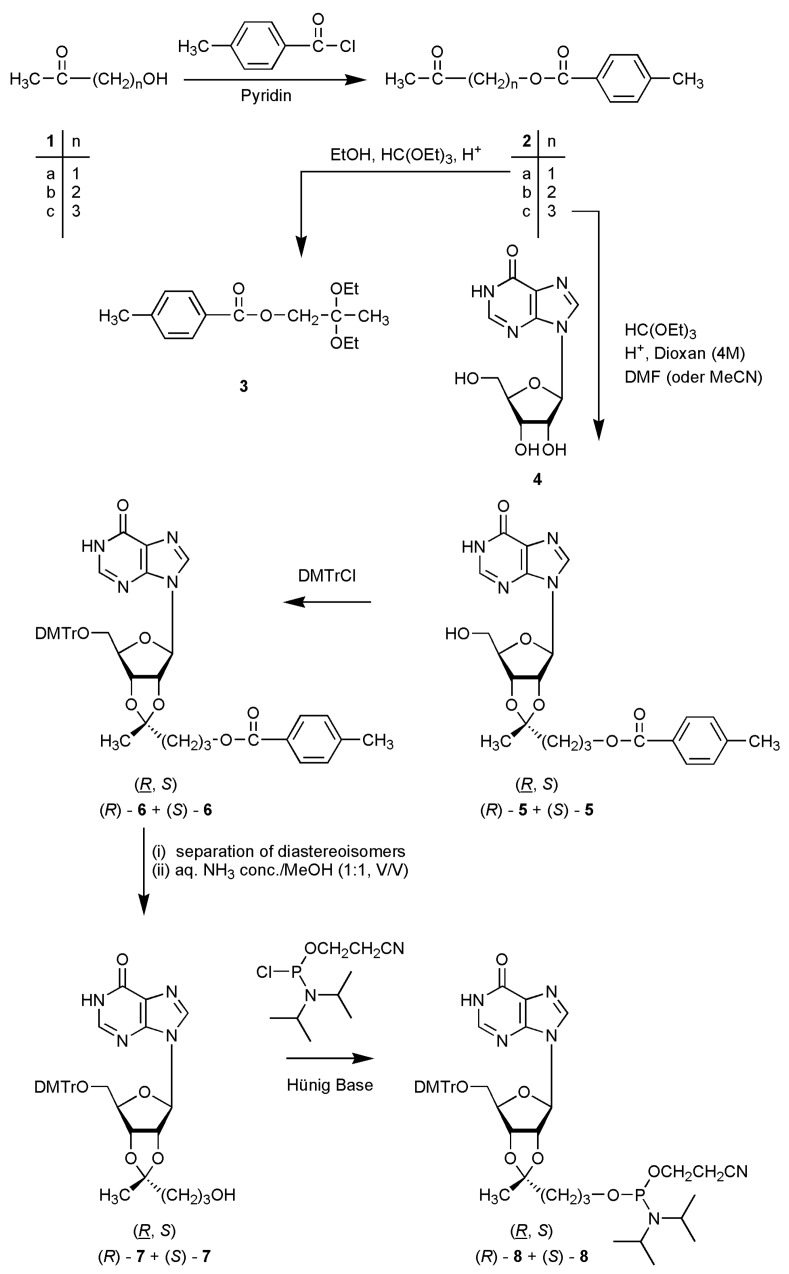
Synthesis of the target structures. In case of the ketals (**5**–**8**) the *R* diastereoisomers always are shown.

It can be seen that in case of incorporation of either one or two modified units into an inosine trimer the resulting log*P* value depends on the position of incorporation: Substitution of one inosine by an inosine ketal at the 3’-end results in an oligomer (entry 2, Table 1) with a free primary hydroxyl group at the 5’-terminus; this oligomer shows a significantly lower lipophilicity than the corresponding trimers carrying the modified inosine derivative either in the middle position (entry 3) or at the 5’-end (entry 4). An analogous result can be seen for trimers carrying two modifications (entries 5-7, Table 1). The synthesis of 2’-cyanoethyl phosphoramidite building blocks of inosine O-2’,3’-cyclic ketals is shown in Scheme 1. First, we converted the (ω-1) ketoalcohols **1a**-**c **to the tolyl-protected compounds **2a**-**c. **Exemplary, Figure 4 displays the three-dimensional structure of **2b** obtained from an X-ray analysis [7].

**Table 1:** Calculatedlog *P* values of inosine oligonucleotide trimers as a function of the number and position of inosine O-2’,3’-cyclic ketal derivatives (see Figure 2).

Entry	Compound	Number of modified units	Calculated log*P* Values
1	5’-d(IpIpI)	0	-6.67 ± 1.06
2	5’-d(IpIpI*)	1	-5.29 ± 1.12
3	5’-d(IpI*pI)	1	-4.29 ± 1.16
4	5’-d(I*pIpI)	1	-4.29 ± 1.16
5	5’-d(IpI*pI*)	2	-2.92 ± 1.22
6	5’-d(I*pIpI*)	2	-2.92 ± 1.22
7	5’-d(I*pI*pI)	2	-1.91 ± 1.29
8	5’-(I*pI*pI*)	3	-0.54 ± 1.32

Earlier, it had been shown that reaction of inosine (**4**) with (ω-1) ketoesters such as ethyl levulinate or unsymmetrical ketones such as pentan-2-one leads to O-2’,3’-ketals with predominant or even exclusive formation of the (*R*) configuration at the newly formed stereogenic center [8,9,10,11,12] [the (*R*)- and (*S*)-notation within this manuscript refers always to the configuration at the stereogenic center of the ketal moiety]. We now prepared the keto esters **2a**-**c**, of which only compound **2c** had been described earlier [13]. All attempts to react inosine with compound **2a** in the presence of triethyl orthoformate and 4M HCl in 1,4-dioxane in various solvents failed. Next, we, therefore, converted compound **2a** into the open acetal **3.** A subsequent transacetalisation with inosine (**4**) which usually occurs under mild reaction conditions also failed. Also ketalisation of inosine with the elongated (ω-1) ketoester **2b** did not show the desired result. The reason for this result might be an electrostatic repulsion of the ester oxygen atom and the ribose oxygen.

At least, reaction of **4** with 4-oxopentyl-4-methylbenzoate, (**2c**) under the reaction conditions mentioned above gave the desired product **5**, however, ^1^H- and ^13^C-NMR spectroscopy proved the formation of a diastereoisomeric mixture [(*R*)-**5** + (*S*)-**5**]. Integration of the ^1^H-NMR resonances of the clearly separated ketal Me groups indicated a ratio of 47% of the (*R*)- and 53% of the (*S*)-diastereoisomer. The assignment of the NMR resonances of the different methyl groups as well as of other signals was made on the basis of a comparison with the ^1^H- and ^13^C-NMR spectra of (*R*)-2’,3’-O-(3-carboxy-1-methylpropyliden)adenosine from which an X-ray analysis had been performed earlier [14] as well as by gradient-selected homo- and heteronuclear correlation spectroscopy. 

A chromatographic separation of the mixture (*R*)-**5** + (*S*)-**5** proved extremely difficult; only a TLC with a 20-fold development in EtOAc/toluene (97:3, v/v) showed the presence of two products. Because the positioning of the ketal side chain is of decisive influence on the topology of oligonucleotides carrying such modified building blocks, a separation of the diastereoisomers is at least inevitable. Next, we converted the mixture (*R*)-**5** + (*S*)-**5 **into the 4,4’-dimethoxytriphenylmethyl derivatives (*R*)-**6** + (*S*)-**6. **On this stage the diastereoisomeric mixture could be separated silica gel chromatographically by elution with EtOAc/toluene (97:3, v/v). Both diastereoisomers were characterized by ^1^H-NMR spectra. 

In order to prove if a chromatographic separation is also possible on the stage of the de-toluoylated compounds a mixture of (*R*)-**6** + (*S*)-**6 **was deprotected by treatment with conc. aq. ammonia/MeOH (1:1, v/v). After 72 h a mixture of (*R*)-**7** + (*S*)-**7** was isolated in moderate 38% yield. As it was found that a chromatographic separation of the diastereoisomers was very difficult, the separation was performed on the stage of compounds **6**. De-toluoylation was performed on either the stage of a diastereoisomeric mixture or on the stage of the separated isomers and afforded (*R*)-**7** and (*S*)-**7. **A first subsequent phosphitylation [15] of a mixture of (*R*)-**7** + (*S*)-**7 **with chloro-(2-cyanoethoxy)-N,N-diisopropylethylaminophosphane (CH_2_Cl_2_, Hünig’s base, 3 h) gave, after chromatography, a mixture of four diastereoisomers (additional R*_P_*and S*_P_* diastereoisomers) in 67% total yield. The phosphitylation of the separated (*R*)-**7** and (*S*)-**7 **isomers, their incorporation into oligonucleotides as well as the base pairing properties of such oligomers will be published in following manuscripts. We anticipate that appending of the novel building blocks to one or both termini of an unmodified oligonucleotide will lead to gap-mers with unaltered binding to their complementary strands but that their incorporation into the innermost part of a nucleic acid will give new and autonomous nucleic acid pairing systems.

## Experimental

### General

Thin-layer chromatography (TLC): Silica gel 60 F_254_ plates (VWR, Darmstadt, Germany). UV-Spectroscopy: U-3200 spectrophotometer (Hitachi, Japan); λ_max_ in nm; ε in M^-1^cm^-1^. NMR Spectra were recorded on AC-250 and AMX-500 spectrometers (Bruker BioSpin, Rheinstetten , Germany). Operational frequencies: ^1^H-NMR: 250.13, 500.14 MHz; ^13^C-NMR: 62.896, 125.700 MHz. Chemical shifts (δ values) are in parts per million relative to tetramethylsilane as internal standard. Microanalyses were performed by Mikroanalytisches Labor Beller-Matthies (Göttingen, Germany). Melting points were measured on a Büchi SMP 20 apparatus and are not corrected. The 3D-optimized structures were obtained using the program *ChemSketch/3D Viewer*, version 12.0, from Advanced Chemistry Development Inc., Toronto, Canada (http://www.acdlabs.com).

### 2-Oxopropyl-4-methylbenzoate (**2a**)

3-Hydroxopropan-2-one (**1a**, 1.13 g, 15 mmol) was dissolved in anhydr. pyridine (3 mL) and cooled in an ice bath. After drop-wise addition of tolyl chloride (1.55 g, 10 mmol) the mixture was stirred for 30 min, warmed up to ambient temperature and stirred overnight. Then, the reaction mixture was poured into ice-water (100 mL), acidified by addition of conc. hydrochloric acid and warmed up to ambient temperature. After extraction with CHCl_3_ the organic layer was separated and washed with 5% aqueous NaHCO_3_. The organic phase was dried (Na_2_SO_4_) and evaporated. After drying under high vacuum 1.7 g (88%) of the title compound were obtained as colourless crystals. M.p. 48 °C; TLC (silica gel, EtOAc/n-hexane, 33:67, v/v): *R*_f_, 0.7; Anal. calcd. for C_11_H_12_O_3_ (192.211): C, 68.74; H, 6.29. Found: C, 68.65; H, 6.18; ^1^H-NMR (d_6_-DMSO): δ, 2.16 (3H, s, CH_3_-arom.); 2.40 (3H, s, CH_3_-C=O); 4.99 (2H, s, CH_2_); 7.35, 7.89 (2 × 2H, d, *J* = 7.5 Hz, H-arom.); ^13^C-NMR (d_6_-DMSO): δ, 21.6 (CH_3_-arom.); 26.4 (CH_3_-C=O); 69.1 (CH_2_); 126.9, 129.80, 144.5 (4 C-arom.); 165.6 (C=O-ester); 202.3 [C(=O)-CH_3_].

### 2,2-Diethoxypropyl-4-methylbenzoate (**3**)

To compound **2a** (0.96 g, 5 mmol) was added triethyl orthoformate (0.82 g, 5.5 mmol), anhydrous ethanol (0.23 g, 5 mmol) and one drop of conc. sulphuric acid. After stirring at room temperature overnight the mixture was evaporated and chromatographed on silica gel 60 (6 × 10 cm, EtOAc/n-hexane, 33:67, v/v). From the main fraction the title compound was obtained as a colourless foam upon evaporation. Yield: 0.43 g (32%). TLC (silica gel, EtOAc/n-hexane, 33:67, v/v): *R*_f_, 0.95; ^1^H-NMR (d_6_-DMSO): δ, 1.10 (3H, t, *J* = 7.5 Hz, CH_3_-ethyl); 1.36 [CH_3_-C(OEt)_2_]; 2.50 (3H, s, CH_3_-arom.); 3.47 (2H, q, *J* = 7.5 Hz, CH_2_-ethyl); 4.22 (2H, s, CH_2_); 7.34, 7.88 (2 × 2H, d, *J* = 7.5 Hz, H-arom.); ^13^C-NMR (d_6_-DMSO): δ, 15.8 (2 CH_3_CH_2_O); 21.6 (2 CH_3_); 56.1 (2 CH_3_CH_2_O); 65.3 (CH_2_); 127.2, 129.8, 144.3 (4 C-arom.); 99.4 (C-ketal); 165.7 (C=O-ester).

### 3-Oxobutyl-4-methylbenzoate (**2b**)

4-Hydroxybutan-2-one (**1b**, 1.32 g, 15 mmol) was reacted with tolyl chloride (1.55 g, 10 mmol) and worked up as described for **2a**. After crystallization from hot propan-2-ol colourless crystals of **2b** (1.55 g, 75%) were obtained. M.p. 37 °C; TLC (silica gel, EtOAc/n-hexane, 33:67, v/v): *R*_f_, 0.7; Anal. calcd. for C_12_H_14_O_3_ (206.238): C, 69.88; H, 6.84. Found: C, 69.74; H, 6.65; ^1^H-NMR (d_6_-DMSO): δ, 2.10 (3H, s, CH_3_-arom.); 2.32 (3H, s, CH_3_-C=O); 2.87 (2H, t, *J* = 5.5 Hz, CH_2_-C=O); 4.36 (2H, t, *J* = 7.5 Hz, CH_2_-O); 7.25, 7.74 (2 2H, d, *J* = 7.3 Hz, H-arom.); ^13^C-NMR (d_6_-DMSO): δ, 21.6 (CH_3_-arom.); 30.4 (CH_3_-C=O); 42.1 (CH_2_-C=O); 60.2 (CH_2_-O); 127.4, 129.7, 144.1 (4 C-arom.); 166.1 (C=O-ester); 208.2 [C(=O)-CH_3_].

### 4-Oxopentyl 4-methylbenzoate (**2c**) [13]

5-Hydroxypentan-2-one (**1c**, 1.53 g, 15 mmol) was reacted with tolyl chloride (1.55 g, 10 mmol) and worked up as described for **2a. **After column chromatography (silica gel 60H, 6 × 10 cm, EtOAc/n-hexane, 33:67, v/v) the main fraction was pooled and evaporated to dryness to give a colourless oil of **2c** (1.72 g, 78%). TLC (silica gel, EtOAc/n-hexane, 33:67, v/v): *R*_f_, 0.68; Anal. calcd. for C_13_H_16_O_3 _(220.264): C, 70.89, H, 7.32. Found: C, 70.78; H, 7.21; ^1^H-NMR (d_6_-DMSO): δ, 1.89 (2H, pq, *J* = 7.5 Hz, CH_2_); 2.10 (3H, s, CH_3_-C=O); 2.38 (3H, s, CH_3_-arom.); 2.60 (2H, t, *J* = 7.5 Hz, CH_2_-C=O); 4.21 (2H, t, *J* = 7.5 Hz, CH_2_-O); 7.32, 7.84 (2 × 2H, d, *J* = 7.5 Hz, H-arom.); ^13^C-NMR (d_6_-DMSO): δ, 21.6 (CH_2_); 23.0 (CH_3_-arom.); 30.2 [CH_3_-C(=O)]; 40.3 [CH_2_-C(=O)]; 64.4 (CH_2_-O); 127.5, 129.7, 144.0 (4 C-arom.); 166.2 (C=O-ester); 208.2 [C(=O)-CH_3_].

### 3-((3aS,4R,6R,6aS)-4-(hydroxymethyl)-2-methyl-6-(6-oxo-1,6-dihydropurin-9-yl)-tetrahydrofuro[3,4-d][1,3]dioxol-2-yl)propyl-4-methylbenzoate, diastereoisomeric mixture **[(R)-5 + (S)-5**]

Anhydrous inosine (**4**, 0.54 g, 2 mmol) was suspended in anhydrous MeCN (7.5 mL), and compound **2c** (2.2 g, 10 mmol) and triethyl orthoformate (0.5 ml, 3 mmol) were added. Upon drop-wise addition of 4M HCl in 1,4-dioxane (1.75 mL) the inosine went into solution. The reaction mixture was stirred at ambient temperature for 24 h and then evaporated to dryness. Column chromatography (silica gel 60, 6 × 15 cm, stepwise elution: (i) CHCl_3_/MeOH, 97:3, 250 ml; (ii) CHCl_3_/MeOH, 9:1, 250 ml, v/v, each) afforded one main fraction from which a diastereoisomeric mixture of compound **5** (0.56 g, 60%) was obtained after evaporation of the solvent as a colourless solid. M.p. 229 °C (decomp.); TLC (silica gel, CHCl_3_/MeOH, 9:1, v/v): *R*_f_, 0.62; UV (MeOH): λ_max_, 248 nm (ε = 28,200 M^-1^cm^-1^; ε_260_ = 18,200 M^-1^cm^-1^); Anal. calcd. for C_23_H_26_N_4_O_7_ (470.514): C, 58.72%; H, 5.57%; N, 11.91%. Found: C, 58.80%, H, 5.49%; N, 11.81%; ^1^H-NMR (d_6_-DMSO): δ, 12.5 (1H, br., s, NH); 8.3002 and 8.2970 (2 × 1H, 2 s, H-2); 8.0731 and 8.0668 (2 × 1H, 2 s, H-8); 7.86 and 7.83 (2 × 2H, 2 d, *J* = 5.0 Hz, H-arom.), 7.34 and 7.30 (2 × 2H, 2 d, *J* = 5.0 Hz, H-arom.); 6.14 (2 × 1H, 2 d, *J*(1’,2’) = 2.5 Hz, H-1’); 5.341-5.359 and 5.304-5.286 (2 × 1H, 2 × dd, *J*(2’,1’) = 2.5 Hz, *J*(2’,3’) = 6.5 Hz, H-2’); 5.10 (1H, m, 5’-OH); 4.994-4.956 (2 × 1H, 2 × dd, *J*(3’,2’) = 6.5 Hz), *J*(3’,4’) = 2.5 Hz, H-3’); 4.33 (1H, m, H-4’); 4.255-4.235 (2H, m, CH_2_-O-C(O)); 3.52 (2H, m, H_2_-5’); 2.39 and 2.38 (2 × 3H, 2 × s, CH_3_-arom.); 1.93 (2H, m, CH_2_); 1.75 (2H, m, CH_2_); 1.538 (3H, s, CH_3_-ketal (R)); 1.333 (3H, s, CH_3_-ketal (S)); ^13^C-NMR (d_6_-DMSO): δ, 146.5 (C-2); 148.2 (C-4); 115.2 (C-5); 157.0 (C-6); 144.0 (C-8); 87.4 (C-1’); 84.3 and 84.7 (C-2’); 81.7 and 82.1 (C-3’); 90.0 (C-4’); 61.9 (C-5’); 23.2 and 23.9 (CH_3_-ketal); 114.8 (C-quart. ketal); 35.0 and 35.6 (CH_2_); 21.6 (CH_2_); 64.8 (CH_2_-O); 166.2 (C(=O)-ester); 124.9, 129.6, 127.6 and 139.2 (C-arom.); 25.5 (CH_3_-arom.).

### 3-((3aS,4R,6R,6aS)-4-(bis(4-methoxyphenyl)(phenyl)methoxy)-methyl)-2-methyl-6-(6-oxo-1,6-dihydro-purin-9-yl)-tetrahydrofuro[3,4-d][1,3]dioxol-2-yl)propyl 4-methylbenzoate, diastereoisomeric mixture **[(R)-6 + (S)-6**]

The diastereoisomeric mixture (*R*)-**5** + (*S*)-**5** (470 mg, 1.0 mmol) was dried by repeated co-evaporation from anhydrous pyridine. The residue was dissolved in anhydrous pyridine (6 mL), 4,4’-dimethoxytriphenylmethyl chloride (643 mg, 1.9 mmol) was added, and the reaction mixture was stirred for 3 h under a N_2_ atmosphere. Then, 5% aq. NaHCO_3_ (30 ml) was added, and the mixture was extracted three times with CHCl_3_ (30 mL, each). The combined organic layers were dried (Na_2_SO_4_) and evaporated to dryness (high vacuum). Column chromatography (silica gel 60, 6 × 20 cm, CHCl_3_/MeOH, 97:3, v/v) afforded one main fraction from which the title compound was obtained as colourless glassy foam (0.41 g, 53%). TLC (silica gel, CHCl_3_/MeOH, 97:3, v/v): *R*_f_, 0.38; UV (MeOH): λ_max_, 242 nm (ε = 47,300 M^-1^cm^-1^; ε_260_ = 26,000 M^-1^cm^-1^); Anal. calcd. for C_44_H_44_N_4_O_9_ (772.842): C, 68.38%; H, 5.74%; N, 7.25%. Found: C, 68.45; H, 5.84; N, 7.09. The diastereoisomeric mixture [(*R*)-**6 + **(*S*)-**6**] was separated by column chromatography (silica gel 60, 6 × 25 cm, EtOAc/toluene, 98:2, v/v).

### 3-((2R,3aS,4R,6R,6aS)-4-(bis(4-methoxyphenyl)(phenyl)methoxy)-methyl)-2-methyl-6-(6-oxo-1,6-dihydropurin-9-yl)-tetrahydrofuro[3,4-d][1,3]dioxol-2-yl)propyl 4-methylbenzoate **[(R)-6]**

From the slower migrating zone the (*R*) diastereoisomer was isolated as colourless foam. TLC (silica gel, EtOAc/toluene, 98:2, v/v): *R*_f_, 0.45; ^1^H-NMR (d_6_-DMSO): δ, 13.23 (1H, s, NH); 8.16 (1H, s, H-2); 7.94 (1H, s, H-8); 8.00 (2 × 1H, d, *J* = 7.5 Hz, DMTr); 7.40-7.19 (13 H, m, DMT); 6.81 (2 × 2H, H-arom.); 6.17 (1H, d, *J*(1’,2’) = 2.5 Hz, H-1’); 5.46 (1H, dd, *J*(2’,1’) = 2.5 Hz, *J*(2’,3’) = 5.0 Hz, H-2’); 5.00 (1H, dd, *J*(3’,2’) = 5.0 Hz, *J*(3’,4’) = 3.0 Hz, H-3’); 4.61 (1H, m, H-4’); 4.44 (2H, m, H_2_-5’); 3.78 (6H, s, 2 × OCH_3_); 3.33 (2H, m, CH_2_); 2.44 (3H, s, CH_3_-arom.); 1.43 (2H, m, CH_2_); 1.30 (3H, s, CH_3_-ketal).

### 3-((2S,3aS,4R,6R,6aS)-4-(bis((4-methoxyphenyl)(phenyl)methoxy)-methyl)-2-methyl-6-(6-oxo-1,6-dihydropurin-9-yl)-tetrahydrofuro[3,4-d][1,3]dioxol-2-yl)propyl 4-methylbenzoate **[(S)-6]**

From the faster migrating zone the (*S*) diastereoisomer was isolated as colourless foam. TLC (silica gel, EtOAc/toluene, 98:2, v/v): *R*_f_, 0.55); ^1^H-NMR (d_6_-DMSO): δ, 12.83 (1H, s, NH); 7.97 (1H, s, H-2); 7.90 (1H, s, H-8); 7.36-7.22 (13H, m, DMT); 6.80-6.77 (4H, m, H-arom.); 6.16 (1H, m, H-1’); 5.37 (1H, d, *J*(2’,3’) = 7.5 Hz, H-2’); 4.99 (1H, d, *J*(3’,2’) = 7.5 Hz, H-3’); 4.57 (1H, m, H-4’); 4.35 (2H, m, H_2_-5’); 3.78 (6H, s, 2 × OCH_3_); 3.33 (2H, m, CH_2_); 2.41 (3H, m, CH_3_-arom.); 1.85 (2H, m, CH_2_); 1.65 (3H, s, CH_3_-acetal); 1.29 (2H, m, CH_2_).

### 3-((3aS,4R,6R,6aS)-6-((bis(4-methoxyphenyl)(phenyl)methoxy)methyl)-2-(3-hydroxypropyl)-2-methyl-tetrahydrofuro[3,4-d][1,3]dioxol-4-yl)-1H-purin-6(9H)-one, diastereoisomeric mixture **[(R)-7 + (S)-7**]

A diastereoisomeric mixture of (*R*)-**6 **+ (*S*)-**6** (772 mg, 1.0 mmol) was dissolved in 7M NH_3_ in MeOH (20 mL) and stirred for 72 h at ambient temperature. After evaporation of the solvent the raw product was chromatographed (silica gel 60, (i) CHCl_3_/MeOH, 97:3, (ii) CHCl_3_/MeOH, 9:1, v/v). From the main fraction the title compound [(*R*)-**7 + **(*S*)-**7**] was obtained as colourless solid (0.25 g, 38%). TLC (silica gel, CHCl_3_/MeOH, 9:1, v/v): R_f_, 0.62; UV (MeOH): λ_max_, 240 nm (ε = 38,100 M^-1^cm^-1^; ε_260_ = 23,000 M^-1^cm^-1^); Anal. calcd. for C_36_H_38_N_4_O_8_ (654.709): C, 66.04%; H, 5.85%; N, 8.56%. Found: C, 66.09%; H, 5.91%; N, 8.41%. The diastereoisomeric mixture [(*R*)-**7** + (*S*)-**7**] proved to be chromatographically separable only with difficulties; therefore, the precursor diastereoisomers (*R*)-**6 **and****(*S*)-**6 **were de-toluoylated separately and worked up as described above. 

(*R*)-**7**: ^1^H-NMR (d_6_-DMSO): δ, 12.34 (s, br., 1H, NH); 8.22 (s, 1H, H-2); 7.93 (s, 1H, H-8); 7.286-6.763 (4m, 13H, H-aromat.); 6.20 (d, 1H, *J*(1’,2’) = 1.0 Hz), H-1’); 5.39 (dd, 1H, *J*(2’,1’) = 1.0 Hz, *J*(2’,3’) = 6.5 Hz), H-2’); 4.91 (dd, 1H, *J*(3’,2’) = 6.5 Hz, *J*(3’,4’) = 3.0 Hz, H-3’); 4.50 (1, 1H, *J* = 5.5 Hz, OH); 4.32 (pq, 1H, *J*(4’,5’) = 3.0 Hz, H-4’); 3.73, 3.72 (2s, 2 × 3H, 2 OCH_3_); ~3.4 (m, CH_2_); 3.22, 3.04 (2m, 2H, H_2_-5’); 1.78 (m, 2H, CH_2_); 1.61 (m, 2H, CH_2_); 1.27 (s, 3H, CH_3_-ketal).

(*S*)-**7**:****12.4 (s, br. 1H, NH);****8.24 (s, 1H, H-2); 7.95 (s, 1H, H-8); 7.287-6.762 (4m, 13H, H-aromat.); 6.25 (d, 1H, H-1’); 5.34 (d, 1H, *J*(2’,1’) = 5.0 Hz, H-2’); 4.92 (m, 1H, H-3’); 4.50 (m, 1H, OH); 4.35 (m, 1H, H-4’); 3.45 (m, 2H, CH_2_); 3.724, 3.723 (2s, 2 × 3H, 2 OCH_3_); 3.23, 3.07 (2 m, 2H, H_2_-5’); 1.59 (m, 2H, CH_2_); 1.44 (m, 2H, CH_2_); 1.49 (s, 3H, CH_3_-ketal). 

### 3-((3aS,4R,6R,6aS)-4-((bis(4-methoxyphenyl)(phenyl)-methoxy)methyl)-2-methyl-6-(6-oxo-1,6-dihydropurin-9-yl)-tetrahydrofuro[3,4-d][1,3]dioxol-2-yl)propyl 2-cyanoethyldiisopropyl-phosphoramidite, diastereoisomeric mixture [**(R)-8 + (S)-8**]

The diastereoisomeric mixture (*R*)-**7 + **(*S*)-**7 **(115 mg, 0.175 mmol) was suspended in abs. CH_2_Cl_2_ (7 mL) and *N*-ethyldiisopropylamine (345 µL, 1.97 mmol) as well as chloro-(2-cyanoethoxy)-*N,N*-diisopropylethylaminophosphane (340 µL, 1.53 mmol) were added. After 3 h at ambient temperature (N_2_ atmosphere). The reaction mixture was chromatographed (silica gel 60, 6 × 10 cm, CHCl_3_/MeOH, 9:1, v/v), and the content of the main fraction was pooled. Evaporation of the solvent gave the title phosphoramidites as colourless foam (0.10 g, 67%). TLC (silica gel, CHCl_3_/MeOH, 9:1, v/v): R_f_, 0.50, 0.54, 0.58, 0.61 (4 diastereoisomers); ^31^P-NMR (CDCl_3_): δ, 147.44, 147.36, 139.79, 139.74.

## Conclusions

We have prepared the first phosphoramidite building blocks for automated nucleic acids synthesis which can be incorporated into a growing nucleic acid chain at any position. The novel units hydrophobize the DNA or RNA oligomers stepwise. Furthermore, using either the (R)- or the (S)-diastereoisomer of such a novel building block opens the possibility to place a kink within the nucleic acid at a predetermined position and to expose therewith a certain part of the oligomer. 
